# Towards Long‐Term Stable Perovskite Solar Cells: Degradation Mechanisms and Stabilization Techniques

**DOI:** 10.1002/advs.202306110

**Published:** 2023-11-23

**Authors:** Namyoung Ahn, Mansoo Choi

**Affiliations:** ^1^ Chemistry Division Los Alamos National Laboratory Los Alamos NM 87544 USA; ^2^ Global Frontier Center for Multiscale Energy Systems Seoul National University Seoul 08826 Republic of Korea; ^3^ Department of Mechanical Engineering Seoul National University Seoul 08826 Republic of Korea

**Keywords:** perovskite solar cells, stability, trapped charges, ion instability

## Abstract

It is certain that perovskite materials must be a game‐changer in the solar industry as long as their stability reaches a level comparable with the lifetime of a commercialized Si photovoltaic. However, the operational stability of perovskite solar cells and modules still remains unresolved, especially when devices operate in practical energy‐harvesting modes represented by maximum power point tracking under 1 sun illumination at ambient conditions. This review article covers from fundamental aspects of perovskite instability including chemical decomposition pathways under light soaking and electrical bias, to recent advances and techniques that effectively prevent such degradation of perovskite solar cells and modules. In particular, fundamental causes for permanent degradation due to ion migration and trapped charges are overviewed and explain their interplay between ions and charges. Based on the degradation mechanism, recent advances on the strategies are discussed to slow down the degradation during operation for a practical use of perovskite‐based solar devices.

## Graphical abstract

The operational stability of perovskite solar cells and modules is a key challenge which still remain unresolved. This review article discusses fundamentals of perovskite degradation due to different stresses including air molecules, light illumination, and electrical bias. The operational stability of these devices and recent advances to stabilize devices are overviewed on a way toward commercialization of perovskite solar devices.

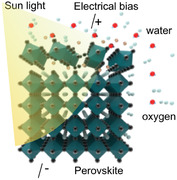

## 1 Introduction

Organic‐inorganic hybrid perovskite has been mainstream among next‐generation solar materials thanks to the excellent certified power conversion efficiency (PCE) exceeding 25%.^[^ ^1^ ^]^ The record‐breaking has not been discontinued since the official certification started in 2013, indicating that it may be just a matter of time to outcompete the world best PCE (26.8%, Silicon heterostructures) of conventional crystalline Si cells.^[^ ^2^ ^]^ Very recently, Shockley–Queisser limit for silicon has been overcome via perovskite/Si tandem cells exhibiting over 33%.^[^ ^2^, ^3^ ^]^ Above its incredible performance skyrocketing, these technologies bring many of new applications, which include flexible,^[^ ^4^ ^]^ wearable,^[^ ^5^ ^]^ lightweight,^[^ ^6^ ^]^ and portable energy harvesting devices,^[^ ^7^ ^]^ as well as building‐integrated photovoltaics (BIPVs).^[^ ^8^ ^]^ Indeed, solar industry will be poised to replace conventional light absorbers with emerging perovskite materials for the bull markets growing in the context of the global corporate renewable energy initiative (RE100).^[^ ^9^ ^]^

To realize the commercialization of perovskite‐based solar devices in any forms of applications, the most critical hurdle will arise from the low stability of perovskite,^[^ ^10^ ^]^ especially in cases where the devices operate in energy‐harvesting modes because these perovskites show much faster degradation under light illumination and electrical bias.^[^ ^11^ ^]^ In that sense, consensus statements for the stability test of perovskite devices were established in 2020 based on the International Summit on Organic PV Stability (ISOS) protocols which included test procedures for light soaking and electrical bias conditions (ISOS‐L).^[^ ^12^ ^]^ When tested based on the ISOS‐L protocol, the operational stability of perovskite devices reported so far has been limited up to a year^[^ ^13^ ^]^ indicating that lifetimes of these solar cells still need to be improved for practical implementation.^[^ ^11^ ^]^

It has been observed that breakdown of perovskite crystals occurs due to various external stresses including humid air,^[^ ^14^ ^]^ heat,^[^ ^15^ ^]^ light,^[^ ^16^ ^]^ and electric field.^[^ ^10^, ^17^ ^]^ These factors synergistically trigger either trapped‐charge driven degradation via chemical reaction in the presence of oxygen (O_2_) and water (H_2_O),^[^ ^18^ ^]^ or ion migration and segregation^[^ ^19^ ^]^ easily occurring through defect sites. While it turned out that material transformation due to the latter effect could be recovered spontaneously by taking advantage of reversible characteristics of ion movements,^[^ ^20^ ^]^ the former mechanism was confirmed to cause irreversible degradation due to charge carriers trapped along grain boundaries by producing stable lead halide (PbX_2_) and even lead hydroxide (PbOH) species.^[^ ^18^, ^21^ ^]^ To suppress charge‐driven degradation under H_2_O and O_2_, encapsulation technique was developed to block those molecules^[^ ^22^ ^]^ However, since it is not possible to perfectly remove the penetration of these molecules, effective mitigation ways of charge accumulation such as reducing defects of grain boundaries in perovskite crystals should be developed to prevent the irreversible degradation and improve the material stability.

When it comes to perovskite solar cells employing charge‐transporting layers (CTLs) and electrodes, causes and pathways of perovskite degradation become more diverse as the whole system is more complicated. For example, a widely used TiO_2_ electron‐transporting layer (ETL) can be excited with UV irradiation, which may possibly cause severe degradation at the interface of TiO_2_/perovskite.^[^ ^23^ ^]^ Additionally, metal ions from a top metal electrode can readily penetrate into the perovskite layer in the presence of electric field, which also leads to a quick performance decay.^[^ ^24^ ^]^ Even if these external causes on degradation are excluded, it has been commonly witnessed that performance degradation is quite rapid under light illumination especially when a forward bias is applied so as to convert solar energy into electricity.^[^ ^25^ ^]^ These imply that real operation conditions give rise to harsh environment for perovskite materials because of charge accumulation at interfaces and vigorous ion migration induced by electric field. Furthermore, both effects can interplay each other to form deep‐level trap (defect) states where nonradiative recombination prevails over radiative recombination or charge collection,^[^ ^26^ ^]^ causing performance drop due to carrier losses^[^ ^27^ ^]^ and irreversible decomposition of the perovskite layer.^[^ ^26^ ^]^

Long‐term stability becomes more challenging in consideration of perovskite modules, as the module system requires post‐treatment such as serial laser scribing^[^ ^28^ ^]^ and bring many difficulties in encapsulation^[^ ^29^ ^]^ and cell‐by‐cell uniformity.^[^ ^30^ ^]^ In particular, the latter effect, so‐called current mismatch, can lead to considerable performance lost, and also unbalanced bias load (for example, reverse biasing on a low current cell and strong forward bias on high current cells).^[^ ^31^ ^]^ This circumstance may lead to even faster performance drop of a perovskite module due to perovskite instability under reverse and strong forward bias conditions.^[^ ^32^ ^]^ As studies on the module stability are relatively lacking, further research on this topic should be done to demonstrate long‐term stable perovskite modules.

The aim of this present review is to overview up‐to‐date studies on the stability of perovskite materials, perovskite solar cells, and modules with a special focus on operational stability for its practical applications. In this respect, we discuss the fundamental mechanisms of perovskite crystal growth and breakdown, main origins for perovskite degradation, and degradation phenomena in perovskite solar cells during operation. Additionally, recent strategies for long‐term operational stability are introduced and discussed together with underlying science.

## 2 Degradation of Perovskite Materials

### 2.1 Mechanism of Perovskite Crystal Growth

Solution‐processable perovskite layers can be fabricated via solution deposition techniques such as spin‐coating,^[^ ^33^ ^]^ blade coating,^[^ ^34^ ^]^ and spray coating.^[^ ^35^ ^]^ Typically, lead halide (PbX_2_) and organic halide (AX) precursors prepared by dissolving them with desired compositions and concentration in organic solvents like dimethylformamide (DMF) and dimethyl sulfoxide (DMSO) are deposited on a substrate by above‐mentioned solution techniques. When solvents are drying after deposition, the crystallization process begins to occur based on thermodynamic principles. It should be noted that the lead halide is less soluble than the organic halide in any solvents, leading to the crystallization of PbX_2_ first and subsequent reaction of solid PbX_2_ and solution‐state AX.^[^ ^33^ ^]^ So, the chemical reaction for perovskite crystallization can be simplified as follows,^[^ ^36^ ^]^

(1) $${\mathrm{PbX}}_{2}\left(s\right)+\mathrm{AX}\left(\mathrm{sol}\right)↔{\mathrm{APbX}}_{3}\left(s\right)$$

where the concentration of AX in a solvent regulates the direction of the chemical reaction. For instance, an AX concentration (*C*) higher than an equilibrium concentration (*C*_0_) of this reaction will lead to the right direction. This reaction mechanism can be more specifically interpreted via the change of Gibbs free energy as suggested in the ref. [37].

In the reaction process, the change in Gibbs free energy per unit volume (*ΔG*_v_) can be given by,^[^ ^38^ ^]^

(2) $$\Delta G_{V}=-\frac{kT}{V_{m}}\ln\frac{C}{C_{0}}$$

where *k*, *T*, and *V*_m_ are the Boltzmann constant, the temperature, and the volume of the solute particle, respectively. One should also consider the Gibbs energy change per unit area (*ΔG*_s_) due to surface tension as the reaction proceeds in solution.

(3) $$\Delta G_{s}=\bar{\sigma_{\mathrm{Sl}}}$$

where $\bar{\sigma_{\mathrm{Sl}}}$ is the averaged surface tension. Therefore, the total change in Gibbs free energy of cubic‐shaped perovskites can be expressed as,

(4) $$\Delta G=a^{3}\;\Delta G_{v}+6a^{2}\Delta G_{s}=-a^{3}\frac{kT}{V_{m}}\ln\frac{C}{C_{0}}+6a^{2}\bar{\sigma_{\mathrm{Sl}}}$$

where *a* is the length of a cubic perovskite. The crystal growth (or breakdown) will occur toward decrease in the Gibbs free energy.

**Figure 1:**
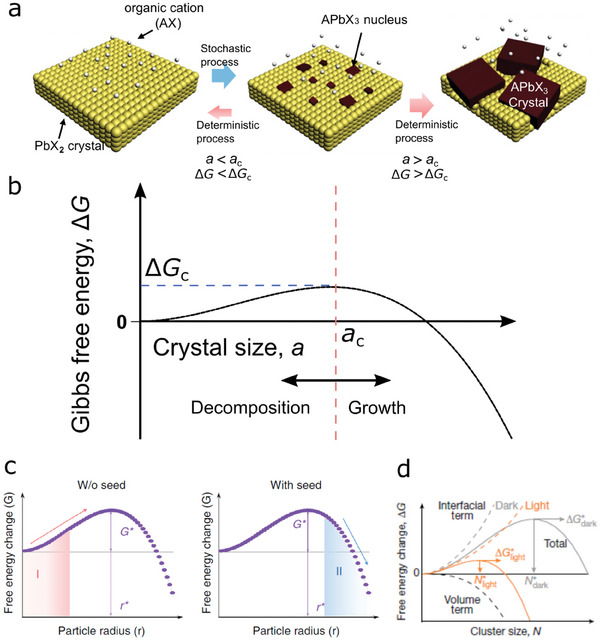
Thermodynamic regulation of perovskite crystal growth. a) Schematic illustration of the growth mechanism of perovskite crystals. Reactions between organic halide (AX) and lead halide (PbX_2_) occurs stochastically, leading to the formation of APbX_3_ nuclei. A nucleus with an energy higher than the critical Gibbs free energy (*ΔG*_c_) can grow until its size reaches the critical size (*a*_c_). The nucleus can further grow spontaneously as long as reactants exist. If an energy of a nucleus does not exceed *ΔG*_c_, that nucleus will disappear spontaneously. b) A theoretical plot for Gibbs free energy (*ΔG*) *vs* crystal size (*a*) which accounts for the deterministic condition of the perovskite crystal growth. Since all reactions proceed in the direction of decreasing the Gibbs free energy, nuclei larger than the critical crystal size (*a*_c_) are seeds for the spontaneous crystal growth. Panels a,b: reproduced with permission from ref. [37]. Copyright 2015 The Royal Society of Chemistry. c) Mechanism of perovskite seeding growth. Without the seed crystal (left), the change in the Gibbs free energy is not energetically favorable until the process overcomes *ΔG*_c_. With the seed crystal embedded in the lead halide film (right), the crystallization process will take place spontaneously due to reduction of *ΔG* with *a* increasing. Panel c: adapted with permission from ref. [40]. Copyright 2018, Springer Nature Limited. d) the Gibbs free energy diagram of a perovskite crystallization process under light soaking. Photo‐generated charges on the surface of lead halide (PbX_2_) reduce free energy due to surface tension, leading to a low critical *ΔG*_c_. A number of seed crystals increases and an average grain size decreases in the case of crystallization under light illumination. Panel d: adapted with permission from ref. [41]. Copyright 2017, Springer Nature Limited.

It can be assumed that the formation of APbX_3_ nuclei randomly happens due to collisions between AX and PbX_2_ molecule.^[^ ^37^ ^]^ (**Figure** **1**a) The nuclei energy (size) distribution will follow Boltzmann energy distribution ($e^{-\frac{E}{kT}}$) based on the stochastic thermodynamic model. Some nuclei with sufficient energy exceeding a critical Gibbs free energy (*ΔG*_c_) can grow spontaneously because the growth reaction is energetically favorable. The critical Gibbs free energy (*ΔG*_c_) can be derived by solving the following equation,

(5) $$\frac{d\Delta G}{da}=3a^{2}\Delta G_{v}+12a\Delta G_{s}=-3a^{2}\frac{kT}{V_{m}}\ln\frac{C}{C_{0}}+12a\;\bar{\sigma_{\mathrm{Sl}}}=0$$

(6) $$∴\;a_{c}=\frac{4\bar{\sigma_{\mathrm{Sl}}}}{\frac{kT}{V_{m}}\ln\frac{C}{C_{0}}},\;\Delta G_{c}=\frac{32{\bar{\sigma_{\mathrm{Sl}}}}^{2}}{{\left(\frac{kT}{V_{m}}\ln\frac{C}{C_{0}}\right)}^{2}}$$

The profile of *ΔG* during crystal growth is shown in Figure 1b. According to thermodynamic modeling, spontaneous crystal growth or breakdown can deterministically occur depending on the energy (or size) of randomly formed nuclei. As inferred from the above Equation (6), a higher concentration (*C*) leads to a low critical Gibbs free energy, allowing for a higher chance of spontaneous growth. This model also indicates that the reaction temperature (*T*), surface tension (*σ*_sl_), and equilibrium concentration (*C*_0_) determines the critical Gibbs free energy.

The studies presented in refs. [37, 39] demonstrated that the size of the methylammonium lead iodide (MAPbI_3_) perovskite crystals fabricated via two‐step spin‐coating method was controlled with the concentration of MAI, and the reaction temperature. The perovskite crystals larger than 600 nm effectively improved photovoltaic performance. In addition, the authors of the study described in ref^[^ ^40^ ^]^ reported that embedding the seed crystal in the lead halide solid led to spontaneous growth of the formamidinium lead iodide (FAPbI_3_) without the critical energy barrier (Figure 1c), thereby enabling the larger crystal size and higher performance. The authors of the study presented in ref^[^ ^41^ ^]^ conducted a theoretical study on crystal growth mechanism under light illumination, in which it was revealed that the crystal size decreased due to light illumination because the critical energy (*ΔG*_c_) was lower by a weak surface tension where the photo‐generated charges existed on the surface of lead halide. (Figure 1d). The mechanism of perovskite crystal growth can be understood quite well using the suggested thermodynamic models as was shown in the above experiments. By taking advantage of the model, one can propose a new fabrication technique to prepare the high‐quality perovskite film.

### 2.2 Instability of Perovskite Materials Due to Moisture and Oxygen

Both the crystal size and crystallinity of perovskite materials is a critical factor in terms of the material stability against chemical environment.^[^ ^42^ ^]^ For the case of a larger crystal with a good single‐crystallinity, chemical reactions between the perovskite crystal and surrounding chemicals (typically H_2_O or O_2_) can be suppressed because the crystal has a small surface‐to‐volume ratio and less penetration pathways for those molecules into the crystal which can trigger the decomposition reaction. To gain simple insight, we can consider a simple situation in which water decomposes perovskite into PbX_2_ solid (not soluble in water) and AX aqueous solution as a reverse reaction introduced in Equation (1).

(7) $${\mathrm{APbX}}_{3}\left(s\right)+H_{2}O\left(l\right)↔{\mathrm{PbX}}_{2}\left(s\right)+\mathrm{AX}\left(\mathrm{aq}\right)$$

The organic halide is very soluble in water like any polar protic solvents, indicating that the equilibrium concentration of the reaction is non‐zero. Since the initial condition of the AX concentration is essentially zero in the APbX_3_ perovskite film, this reaction will go toward the right until it reaches an equilibrium condition spontaneously. It was experimentally confirmed that perovskite films were changed into the yellow PbI_2_ film in dark condition under high humidity. In this respect, the early‐stage studies on the perovskite instability had focused on the material stability against humidity.

In the follow‐up studies presented in ref^[^ ^43^ ^]^, it was observed that water‐induced transformation can be reversibly restored after dehumidification (removal of moisture). Absorption spectra of the restored perovskite film were fully recovered after 6 h of dehumidification even though it was accompanied with a morphological change due to the decomposition and re‐crystallization process. The authors identified that the MAPbI_3_ perovskite could be hydrated through the formation of Lewis‐base adduct. The oxygen atom of the water molecule (Lewis base) is able to form an adduct bonding to Pb atom (Lewis acid), leading to the monohydrate state (CH_3_NH_3_PbI_3_·H_2_O). It can further be hydrated with two water molecules, forming the dihydrate species ((CH_3_NH_3_)_4_PbI_6_·2H_2_O). If more water is added, it leads to the formation of solid PbI_2_ and aqueous CH_3_NH_3_I as we indicated in Equation (7).

On the other hand, moisture‐induced instability for the FA‐based perovskites seems different. In these materials, α‐FAPbI_3_ (black) perovskite experiences rapid phase transition to δ‐phase (yellow) under RH ∼ 80% rather than forming a hydrated state.^[^ ^44^ ^]^ Since the δ‐phase perovskite is no longer able to absorb solar light, it means that the phase transition is very critical to device performance. Still, the film‐grade δ‐phase perovskite can be recovered with dehumidification and heat treatment,^[^ ^44^, ^45^ ^]^ implying that moisture‐induced transformation is reversible for both MA‐ and FA‐based perovskites. In spite of the reversibility of moisture‐induced transformation, it should be prevented due to possible changes in morphologies and crystallinity after recovery. There have been practical approaches to prevent moisture‐induced degradation by blocking direct exposure to moisture via encapsulation techniques and additional hydrophobic treatment. One can take advantage of detachable multifunctional polymer layer with hydrophobicity and anti‐reflection enabled by biomimetic multi‐scale structure approaches as suggested in ref^[^ ^46^ ^]^.

Oxygen molecules also play a critical role in the degradation of perovskite materials regardless of perovskite composition.^[^ ^10b^ ^]^ Although it seemed that only oxygen at room temperature with no light illumination cannot break perovskite crystals, a synergetic effect of oxygen in the presence of light illumination has been experimentally observed.^[^ ^16^, ^47^ ^]^ A recognized mechanism was suggested as photo‐induced formation of superoxide (O_2_^−^) and the resulting deprotonation reaction with organic cations.^[^ ^47b^ ^]^ An ab initio molecular dynamics (AIMD) simulation also confirmed atomistic degradation pathways including the formation of superoxide and breaking of the perovskite crystal induced by the superoxide molecule.^[^ ^48^ ^]^ The oxygen molecules not only affected the perovskite layer, but also possibly oxidated charge‐transporting layers such as Spiro‐MeOTAD.^[^ ^49^ ^]^ The oxidation of charge‐transporting layers led to defect‐mediated recombination losses and prevention of charge separation, significantly reducing photovoltaic performance. Such ingress of oxygen molecule must be mitigated using effective packaging technologies, thereby preventing oxygen‐induced degradation in order to realize long‐term stable perovskite devices.

### 2.3 Ion‐induced Instability: Ion Migration, Segregation, and Amorphization

The perovskite crystal is an ionic crystal having a general chemical formula of ABX_3_, in which the B cation is surrounded by six X anions form BX_6_^−^ octahedrons sitting at the center and A cations sit at the corner of the cube. It was well known that these ionic crystals are intrinsically soft, which led to extensive observations of ion migration, segregation, mixed electronic‐ionic conduction features, and partial amorphization in the family of lead halide perovskites.^[^ ^50^ ^]^ These effects have been also pointed out as a main origin of perovskite instability and anomalous photocurrent–voltage (*I*‐*V*) behaviors such as light‐soaking effect and hysteresis.^[^ ^51^ ^]^

The most recognized mechanism to explain ion migration in MAPbI_3_ perovskites is vacancy‐mediated migration.^[^ ^52^ ^]^ In the crystal, there are different types of point defects (interstitial, vacancies, and substitutions). In particular, vacancies for MA cations (V_MA_^−^) and I anions (V_I_^+^) among those point defects have the lowest formation energies, indicating that those vacancy defects can form spontaneously.^[^ ^53^ ^]^ In this case, MA^+^ cations and I^−^ anions can migrate to adjacent vacancies in the presence of external force. A study presented in ref. [52] conducted theoretical calculation for the activation energy of the vacancy‐mediated migration (*E*_A_), revealing that the *E*_A_ values were 0.58 and 0.84 eV for V_I_^+^ and V_MA_^−^, respectively. These values are low enough to activate ion migration at room temperature especially with an external driving force. It should be noted that the calculated activation energy of V_Pb_^2‐^ is 2.31 eV which is ≈3 to 4 times higher than those of V_I_^+^ and V_MA_^−^.^[^ ^52^ ^]^ In other words, lead cations are not likely responsible for ion migration in this system.

**Figure 2:**
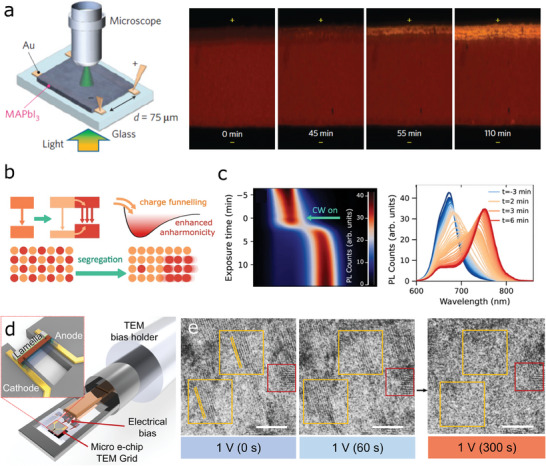
Ion movements in halide perovskite a) an in situ microscopic measurement for ion migration in a MAPbI_3_ film (left) and microscopic images showing real‐time ion migration for 110 min. Adapted with permission from ref. [54b]. Copyright 2014, Springer Nature Limited. b) a schematic illustration of halide segregation c) Direct observation of halide segregation from time evolution of photoluminescence (PL) spectra collected using 400 nm pulsed irradiation. The CW laser turned on at *t* = 0 with 100 W/cm^2^ illumination intensity. Adapted with permission from ref. [61]. Distributed under the CC‐BY 4.0 open access license. d) in situ transmission electron microscopy (TEM) measurement setup with micro e‐chip for applying an electrical bias. The cross‐section of the device was obtained via the focused ion beam (FIB) milling process, which was characterized with TEM e) Observation of partial amorphization under 1V of electrical bias. Specific crystal lattices (110) gradually disappeared for 300 s (yellow box), while the lattice still remained after 300 s. Adapted with permission from ref. [62a]. Copyright 2021, American Chemical Society.

The vacancy‐mediated ion migration has been experimentally confirmed in the presence of electrical biasing.^[^ ^54^ ^]^ The authors of ref. [54b] built a microscopic setup to obtain real‐time optical images of the surface of a perovskite film fabricated on glass with pre‐patterned Au electrodes. (**Figure** **2**a, left) The in situ snapshots obtained for 2 h when 1.2 V µm^−1^ of electric field was applied to the perovskite film showed that the anode side became more transparent as a result of the drift of ions. (Figure 2a, right) It was also confirmed that the ion migration effect varied depending on the grain size. The activation energy (*E*_A_) of ion migration increased as the crystal (grain) became larger, indicating that the lower degree of structural order in the crystal provided ion migration pathways due to the higher densities of defects.

Since ion migration leads to non‐uniform optoelectronic properties across the perovskite layer as a consequence of migrated vacancies and ions, *I*‐*V* characteristics in a perovskite device can experience dramatic changes with the ion migration effect.^[^ ^52^, ^54^, ^55^ ^]^ In general, this results in significant decreases of photovoltaic performance for quite a short time (minute‐scale) by forming ion barriers at the interfaces, by which ion migration has been regarded as a main culprit of fast degradation.^[^ ^56^ ^]^ However, ion migration is reversible as a reverse bias induces opposite direction of ion migration and it was also revealed that performance decay due to ion migration could be restored after dark recovery.^[^ ^20^, ^57^ ^]^ In contrast, the studies described in refs. [58] observed positive effects of ion migration because it can possibly lead to defect annihilation by filling vacancies with interstitial defects.

The perovskite crystal lattice is tolerant to structural distortion even if different cations and anions are mixed together, which allows for the unconstrained stoichiometric engineering.^[^ ^59^ ^]^ This simple approach has made the huge success in terms of both device performance and stability.^[^ ^18^, ^59^ ^]^ However, the mixed perovskites are not free from halide segregation, another type of the ion‐related behavior.^[^ ^60^ ^]^ The mechanism underlying the segregation is still debated, but either photon absorption or current injection seem to be mainly responsible for spatial separation of iodide‐rich and bromide‐rich regions.^[^ ^60^ ^]^ Unfortunately, the effect was associated with the significant decrease in photovoltaic performance as discussed below.^[^ ^60a^ ^]^

Halide segregation has been experimentally observed using various photoluminescence (PL) setups. The clear signature of this effect is the appearance of two‐band PL spectra due to the formation of I‐rich low bandgap phase and Br‐rich high bandgap.^[^ ^60^, ^61^ ^]^ (Figure 2b) These studies presented exhibited that the peak of the PL emission was dramatically shifted with light soaking for a few minutes, indicating that halide segregation occurred very quickly. (Figure 2c) It was experimentally verified that the segregation led to charge funneling into the low‐bandgap region (Figure 2b, lowering the charge extraction efficiency and the open‐circuit voltage.^[^ ^61^ ^]^ Although this directly caused the performance drop in the mixed halide perovskite devices, the segregation‐induced degradation was able to be fully recovered by the re‐mixing process spontaneously forced by entropy.

As discussed earlier, ion movements observed in these perovskites are associated with changes in optoelectronic properties of the material and the resulting performance degradation. The amorphization of perovskite crystals has been also suggested as an intensified effect of ion movements and a cause of huge performance loss.^[^ ^62^ ^]^ Generally, phase transformation and amorphization are observed when some crystalline material is applied to a strong pressure (≈GPa).^[^ ^63^ ^]^ Perovskites also experience amorphization and re‐crystallization induced by pressure,^[^ ^64^ ^]^ indicating that strong ion migration effects might also lead to the amorphization of perovskite. A recent study presented in the ref. [62a] demonstrated an in situ transmission electron microscopy (TEM) setup with a micro e‐chip which enabled real‐time detection of perovskite crystallographic information under electrical biasing. The authors prepared a nano perovskite lamella device prepared via the focused ion beam (FIB) technique and mounted the sample in the setup as shown in Figure 2d. The high‐resolution TEM images acquired for 300 sec under 1 V of electrical bias clearly presented the gradual loss of the crystallinity only for a specific crystal plane (110), while the other lattice remained after the measurement. The authors suggested that the partial amorphization of the (110) crystal plane originated from the iodide migration. Remarkably, the electrically amorphized perovskite was effectively re‐crystallized through heat treatment, indicating that the amorphization process was also in accordance with the ion migration and halide segregation phenomena as all these effects showed reversibility.

Although ion‐induced instability in the perovskites provides interesting observations in the device level, it needs to be taken care to achieve long‐term stability. The reversible characteristics are quite beneficial for long‐term operational stability (which we will discuss in Section 4.1), but the best way to stabilize the device will be to mitigate ion instabilities.

### 2.4 Trapped‐Charge Driven Degradation

Perovskite materials have excellent absorption properties and long carrier lifetime, which make this material a promising light absorber for solar applications.^[^ ^65^ ^]^ Paradoxically, it is known that perovskite light‐absorbing materials are unstable under light illumination.^[^ ^66^ ^]^ One can easily witness that an original black color of a perovskite film or device is rapidly changed to yellow when it stays under light illumination in air condition. Unfortunately, it is impossible to return it to the original black perovskite after the degradation. In other words, the process is an irreversible (permanent) degradation.

It was observed that the irreversible degradation was accelerated by light soaking and exposure to air (H_2_O and O_2_), while the perovskite film was very stable in an inert condition. The study of ref. [16] suggested that the perovskite was quickly degraded due to the presence of superoxide radical (O_2_^−^) generated by interaction between photo‐excited electrons and O_2_ molecules. The effect of charge carriers excited by light absorption on perovskite degradation was intensively investigated to account for light‐induced irreversible degradation.

**Figure 3:**
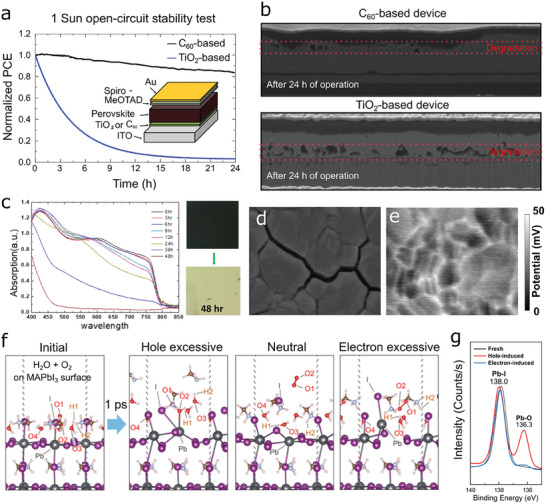
Perovskite degradation induced by charge accumulation a) Device stability test under AM 1.5G 1 sum illumination for perovskite solar cells employing C_60_ (black) and TiO_2_ (blue) as an electron transporting layer (ETL), exhibiting significant differences in performance decay time depending on ETL. b) Morphological changes measured via cross‐sectional scanning electron microscopy (SEM) of the devices shown in panel **a**. Degradation initiated from the TiO_2_/perovskite interface in the case of the TiO_2_‐based device (bottom), while the C_60_‐based device showed relatively slower degradation at the perovskite/Spiro‐MeOTAD interface. c) Evolution of absorption spectra of a perovskite film aging for 48 hours in a customized chamber that electrically deposits N_2_+ corona charges on the film. 90% of relative humidity in the chamber was maintained by continuous water bubbling. Photographic images of the fresh perovskite (right, top) and the aged perovskite (right, bottom) d) Surface morphology of a degraded perovskite film measured via SEM, clearly presenting cracks after degradation were formed along grain boundaries. e) Surface potential of a perovskite film with light soaking measured via Kelvin probe force microscopy (KPFM). Higher potential formed at grain boundaries indicated that charges were accumulated where degradation started in panel d. Panels a‐e: reproduced with permission from ref. [18]. Distributed under the CC‐BY 4.0 open access license. f) ab initio molecular dynamic (AIMD) simulation to investigate degradation reaction between H_2_O, O_2_, and MAPbI_3_ surface depending on different charge state. After 1 ps, +1 charge (hole excessive) and −1 charge (electron excessive) in a MAPbI_3_ crystal unit led to significant deterioration of chemical bonds in the MAPbI_3_ perovskite, while the neutral crystal maintained their structure with no formation of stable chemical bonds between gaseous molecules and MAPbI_3_ components. g) X‐ray photon spectroscopy of the fresh perovskite film and the perovskite films degraded with excessive electrons or holes in air. The Pb‐O bond was observed in both charged films, indicating trapped charges assist the formation of Pb‐O bond and chemical degradation. Panel f‐g: reproduced with permission from ref. [21]. Copyright 2019, The Royal Society of Chemistry.

One interesting observation was that the degradation rates of perovskite solar cells depended on the kind of ETLs (TiO_2_ and C_60_) as shown in **Figure** **3**a.^[^ ^18^ ^]^ The TiO_2_‐based device showed much faster degradation compared to the C_60_‐based device. At the same time, the severe *I‐*‐*V* hysteresis was observed in the TiO_2_‐based device, whereas the C_60_‐based device showed zero hysteresis. This indicated that the instability in the TiO_2_‐based device was also attributed to the huge capacitive effects (charge accumulation). The cross‐sectional morphologies of the degraded devices were additionally characterized using a scanning electron microscope (SEM) combined with a FIB milling, in which the location of the degradation was different depending on the kind of ETLs. (Figure 3b) These results pointed out that the degradation might be triggered where charges were trapped.

To confirm trapped‐charge drive degradation, the authors investigated degradation of the perovskite film in a chamber isolated from outside. Environment was controlled by the gas inlets connected to the gas sources. When a perovskite film was exposed to light and moisture, it showed degradation over time. (Figure 3c) Morphologies of the degraded sample obtained using SEM shown in Figure 3d showed that degradation initiated along grain boundaries where charges were easily trapped as confirmed their kelvin probe force microscopy (KPFM) measurements. (see Figure 3e) Additional experiments to prove trapped‐charge‐driven degradation were conducted by exploiting corona ion deposition, which consistently confirmed that the irreversible degradation occurred due to trapped charges and gaseous molecules (H_2_O and O_2_).

In the follow‐up studies described in refs. [21, 48], the mechanism of trapped charge‐driven degradation was investigated through AIMD computation by tracking the motions of atoms in the presence or absence of the electrostatic charges. The O_2_ and H_2_O molecules sitting on the surface of the MAPbI_3_ crystal tend to actively interact with MA cations and Pb cations only in the presence of the charge regardless of its polarity, while the neutral case does not attract the gaseous molecules and form additional bonding. Intriguingly, the deprotonation of organic cations can be mediated by an iodide anion and a trapped charge as the following procedures,^[^ ^48^ ^]^

(8) $$I^{-}+H_{2}O\to \mathrm{HI}+{\mathrm{OH}}^{-}$$

(9) $${\mathrm{CH}}_{3}{\mathrm{NH}}_{3}^{+}+{\mathrm{OH}}^{-}\to {\mathrm{CH}}_{3}{\mathrm{NH}}_{2}+H_{2}O$$

The deprotonated methylamine (CH_3_NH_2_) is volatile at room temperature, which possibly leads to irreversible chemical reaction (degradation) due to its natural evaporation.^[^ ^67^ ^]^ The O_2_ molecule showed more rigorous attack to break the MAPbI_3_ crystal by directly forming Pb‐O bonds mediated by superoxide (O_2_^−^) generated by the electrostatic charge. These computation results consistently accounted for experimental results regarding trapped‐charge‐driven degradation observed in previous studies.

It also turned out that the polarity of trapped charges also played a critical role in degradation pathways. A study introduced in ref. [21] verified that the degradation rate relied on the polarity of the trapped charges as a result of different degradation mechanisms. In the study, the electron‐rich and hole‐rich MAPbI_3_ layers were realized using the half‐device architecture (the perovskite layer coated on top of the HTL or the ETL) which could selectively extract only one polarity when electron and hole pairs were generated under light illumination. The stability test of these electron‐ and hole‐rich layers showed noticeable differences in their degradation rates depending on the surrounding gaseous conditions. Especially, atmosphere containing both H_2_O and O_2_, the most practical situation, led to extremely fast degradation in the case of hole‐rich perovskites, while the electron‐rich case was quite stable under continuous 1 sun illumination.

To gain insight into polarity‐dependent degradation, the author of the study conducted AIMD calculations for the MAPbI_3_ crystal surrounded by H_2_O and O_2_ molecules. (Figure 3f) The simulation results revealed dissimilar molecular activities depending on the charge polarity for the initial 1 ps. Both neutral and electron‐rich (‐1 charge) cases did not lead to significant breaking of the MAPbI_3_ crystal. On the contrary, in the case of the hole‐rich (+1 charge) MAPbI_3_ slab, the strong and stable Pb‐O bond was formed, and the crystal structure was totally broken after all. The molecular action further led to the formation of Pb‐I‐O‐OH mediated by the deprotonation of CH_3_NH_3_^+^, which gives clear evidence on the fastest degradation observed in the half‐device experiments. The formation of the hydroxide species suggested by AIMD simulations was experimentally confirmed by using X‐ray diffraction (XRD) measurements for the degraded half‐devices. Figure 3g showed X‐ray photoelectron spectroscopic (XPS) results of the fresh and degraded half devices, in which a new peak corresponding to the Pb‐O bond appeared after hole‐induced degradation. These observations indicated that the trapped‐charge‐driven degradation was accelerated by formation of the stable lead hydroxide.

Since trapped charges triggers irreversible degradation of these perovskite materials in the presence of H_2_O and/or O_2_, it will be highly demanded to mitigate charge trapping and block the gaseous molecules as much as possible for their long‐term stability. Charges are easily trapped along grain boundaries where many trap sites exist as well as at interfaces due to surface imperfections and unbalanced charge extraction in a device. Thus, material engineering for less defect sites and larger crystal (high crystallinity) as well as heterostructure engineering for smooth charge collection have been suggested for demonstration of stable perovskite solar cells.

### 2.5 Defect Chemistry and Interplay Between Trapped Charges and ion Migration

The formation of intrinsic defects in crystals is intrinsically motivated for entropic reasons as the mixing of point defects and ions leads to increase in its entropy.^[^ ^68^ ^]^ What is more important is the energy levels of these defects rather than concentrations of point defects. Specifically put, how deep the level of defect state is located compared to the conduction band (CBE) and the valence band edge (VB) will determine impacts on optoelectronic properties and performance.^[^ ^69^ ^]^ It is because the shallow trap states can still lead to radiative recombination while the deep‐level defects are where nonradiative recombination occurs and also provide the sites for long‐lived traps^[^ ^70^ ^]^. The deep‐level states directly lead to significant losses in photovoltaic performance and irreversible degradation due to the charge trapping.^[^ ^71^ ^]^

**Figure 4:**
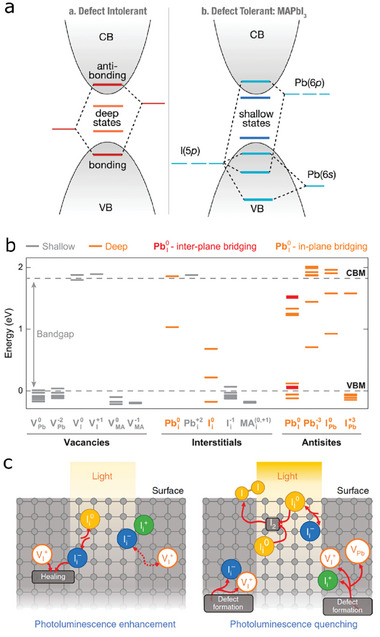
a) An electronic structure of defect‐intolerant semiconductor (e.g. CdSe, GaAs) (left) and defect tolerant perovskite (e.g. MAPbI_3_). If intrinsic defects of a semiconductor compound form deep‐level states in energy bandgap, this material is defect intolerant due to charge trapping. On the contrary, trap‐assisted nonradiative recombination is suppressed in a semiconductor compound where defect energy states are shallow, indicating that optoelectronic properties of perovskite are not subject to defect formation. Adapted with permission from ref. [69b]. Copyright 2014, American Chemical Society. b) Energy levels of defect states of MAPbI_3_ perovskite calculated using the density functional theory (DFT). Energy states of intrinsic defects such as vacancies and iodide interstitials are shallow, which is indicative of defect tolerance. Adapted with permission from ref. [72]. Copyright 2014 American Chemical Society. c) Suggested defect chemistry underlying light‐induced photoluminescence (PL) enhancement and quenching. Trapped charges triggers to produce stable I_2_ species by forming deep‐level defects (neutral I_i_^0^), leading to PL quenching. Adapted with permission from ref. [26]. Copyright 2017, Nature publishing Group.

In this respect, one of superior characteristics of these perovskite materials comes from the defect tolerance, which means that the energy levels of most defects inherently formed in these perovskites are shallow.^[^ ^69b^ ^]^ (**Figure** **4**a) It should be noted that some semiconductors like CdSe and GaAs are defect‐intolerant due to the deep‐level defects. This accounts for excellent photoelectronic performance even if defects are intrinsically formed in the perovskite crystals. The study presented in ref. [72] conducted density functional theory (DFT) calculations in order to obtain the energy states of intrinsic defects. The calculated energy levels are shown in Figure 4b, explaining defect tolerance of the perovskite. As indicated in Section 2.3, the vacancy defects (V_MA_^−^ and V_I_^+^) and interstitial defects (I_i_^−^ and MA_i_^+^) have low formation energies, indicating that defects formed in the perovskite mostly consist of these vacancies and interstitials rather than anti‐site defects with high formation energies. For that reason, the defects in the perovskite do not impair photovoltaic performance, achieving defect tolerance.

When it comes to degradation, high densities of defects may be harmful even if the defects are shallow traps because these shallow traps can be converted into the deep‐level defects through charge‐state transition process due to external forces.^[^ ^26^ ^]^ As discussed in Section 2.3, ions are readily migrated by electric field or light illumination. In some cases, these ion movements may lead to the annihilation of defects (defect healing) of the I_i_^−^V_I_^+^ Frenkel pair. However, a harsh circumstance like strong light illumination rather gives rise to localization of ions and trapping of charge carriers. It may further produce the neutral iodide interstitial and the stable I_2_ in the present of electron and hole trapping as suggested in the study presented in ref. [26].

(10) $$I_{i}^{+}/I_{i}^{-}+1e\to I_{i}^{0}/I_{i}^{-}+1h\to 2I_{i}^{0}\to I_{2}$$

where the $I_{i}^{+}/I_{i}^{-}$ is an iodide interstitial defect pair. The neutral iodide interstitial is a deep‐level defect (see Figure 4b, energy level of I_i_^0^) which acts as the sites for nonradiative recombination, finally causing reduction in PL emissions. (Figure 4c, right). The formation of I_2_ was experimentally confirmed from the degraded MAPbI_3_ using the XRD techniques.^[^ ^18^, ^73^ ^]^

Many other defect‐mediated degradation routes may exist under different circumstances, but the most plausible pathway happening in the halide perovskite will be associated iodide species due to its highest mobility.^[^ ^52^ ^]^ It is noteworthy that the degradation pathway based on defect chemistry includes interplay between ion movements, defect formation, and charge trapping which have been suggested as independent causes of perovskite degradation in previous studies. Their interplay and synergetic effects on degradation play a decisive role in the lifetime of perovskite devices working under light illumination and electric field.

### 2.6 Thermal Stability of Perovskite Materials

Heat is also a critical stress factor to induce degradation of perovskite materials.^[^ ^10b^ ^]^ In an earlier study to investigate heat‐induced degradation, it was observed that MA‐based perovskite films degrade at 85 °C within 24 h even in inert environment, indicating that heat itself could induce degradation of perovskite materials through evaporation of halides and organic cations even in the absence of reactive molecules.^[^ ^74^ ^]^ To overcome the low thermal stability of MA‐based perovskite, compositional engineering by mixing A‐cations^[^ ^75^ ^]^ and halides^[^ ^76^ ^]^, and interfacial treatment^[^ ^77^ ^]^ has been conducted by many research groups. As a result, thermal stability of perovskite solar cells has been greatly improved to a few thousand hours of T_85_ (at 85°C and 50% relative humidity).^[^ ^77^ ^]^ In consideration of the actual operational temperature ranging from −15 to 65 °C, the thermal stability of perovskite films and devices may not be a major issue in terms of perovskite commercialization.

## 3 Operational Stability of Perovskite Solar Cells

In the previous section, we discussed various degradation origins and mechanisms observed in perovskite materials under different circumstances. Moisture and oxygen, ion instabilities, trapped charges, and deep‐level defects have been suggested as main origins of perovskite instabilities. We overview operational stability and degradation mechanisms in complete perovskite solar cells based on knowledge obtained earlier.

### 3.1 Bias‐dependent Degradation Rates in Perovskite Solar Cells

In a solar cell, electricity is generated when photocurrent (*I*_ph_) flows over an electrical barrier formed by an externally applied bias (*V*_applied_). The harvesting power corresponds to *P* = *I*_ph_ *V*_applied_. For the most efficient power harvesting, we need to find a maximum power point (MPP, *P*_max_) and operate a device using a MPP tracking (MPPT) method. To obtain reliable results in terms of operational stability, a perovskite solar cell needs to be tested with a MPPT method under 1 sun light illumination. As we indicated in the introduction section, this procedure is proposed as ISOS‐L protocols for the long‐term stability test.^[^ ^12a^ ^]^

**Figure 5:**
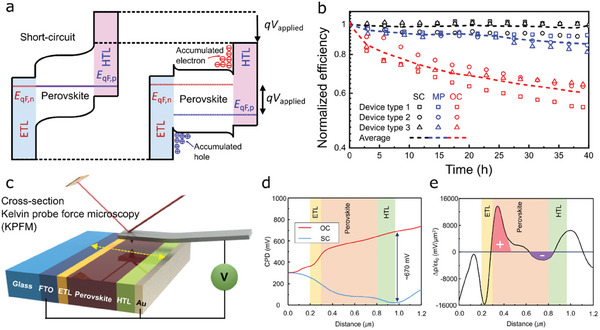
a) A schematic diagram of the electronic band structure of a perovskite solar cell depending on the applied voltage. In the case of the short‐circuit condition (*V*_applied_ = 0 V), fermi levels of ETL, perovskite, and HTL are perfectly aligned. An electrical bias (*V*_applied_) induces splitting of quasi‐fermi levels of the electron in ETL and the hole in HTL, thereby conduction and valence band edges are bent at both interfaces. Photo‐generated charges are localized at the dips induced by electronic band bending. b) Stability test of three perovskite solar cells (glass encapsulated) operated at short circuit (SC), maximum power point tracking (MPPT), and open‐circuit (OC) states under 1 sun illumination. A higher voltage resulted in a lower operation stability due to the effect of charge accumulation. Panels a,b: reproduced with permission from ref. [78a]. Copyright 2022, Elsevier. c) An illustration of cross‐sectional KPFM measurement of a perovskite solar cell. Contact potential differences (CPDs) of a cleaved device are directly probed by applying a bias to a tip and electrodes of the device. d) CPD profiles of a perovskite solar cell employing a TiO_2_ ETL operated at OC (red) and SC (blue) conditions, respectively. e) a profile of density of charges obtained from CPD profiles, indicating localization of holes and electrons at the ETL and HTL interfaces. Panels c‐e: reproduced with permission from ref. [79]. Copyright 2020, American Chemical Society.

The electrical bias is also an important factor to induce electrical stresses to a perovskite device because the electrical barrier not only induces strong electric field inside the device, but also resists flowing of photocurrent, leading to charge accumulation in a device. (**Figure** **5**a) In that sense, the studies described in refs. [78] performed experiments to investigate bias‐dependent stability. The results consistently indicated that a higher electrical bias led to a faster degradation regardless of the device structure. Figure 5b compares the time evolution of normalized efficiencies of three different devices under light illumination depending on electrical conditions.^[^ ^78a^ ^]^ Although the SC condition showed the most stable operation with no stark decrease in its efficiency, the device kept under the OC condition lost 40% of its initial efficiency after 40 h of continuous operation.^[^ ^78a^ ^]^ The efficiency of the device operated by the MPPT method was also decreased over time, but the rate of degradation was much lower than the OC condition. The authors further investigated operational stability for four different biases around MPP, revealing that a higher bias led to a faster degradation. Strikingly, there happened no degradation in the devices operated at biases slightly lower than MPP, indicating that the sufficient extraction of photocurrent helped to boost operational stability.

### 3.2 Electronic Band Bending and Correlated Degradation Due to Accumulated Charges

In a relevant study described in ref. [79], the authors investigated device junction structure and its correlation with degradation of the working perovskite solar cells under 1 sun illumination. It was found that the device heterostructure resulted in the different type of semiconductor junction with different ETLs which was evidenced by e‐beam‐induced current (EBIC) measurements. A device structure consisting of the TiO_2_/perovskite/Spiro‐MeOTAD heterostructure behaved as a dominant p‐n junction solar cell around the TiO_2_/perovskite interface while the C_60_/perovskite/Spiro‐MeOTAD device worked like an ideal p‐i‐n junction. These observations were attributed to a high degree of electronic band bending localized at the specific interface. Additional KPFM measurements for the cross‐sectional plane of the devices shown in Figure 5c provided important information on the potential profile arising from the junction structure. In the dominant p‐n junction structure, it was observed that the potential was predominantly shifted at the corresponding interface (Figure 5d) and the photoexcited charges were accumulated with a significant amount due to the potential barrier. (Figure 5e) It was again confirmed that a lot of charges accumulated at the interface caused fast irreversible degradation as already discussed in Section 2.4. These studies suggest that a proper design of junction structure in perovskite solar cells plays a significant role in realization of the long‐term stable devices. By taking advantage of such heterostructure (or interface) engineering, relevant studies have made great improvements in the device stability when tested based on the ISOS‐L protocol.^[^ ^80^ ^]^ It can be further optimized by demonstrating a perovskite solar cell that can act as an ideal p‐i‐n junction.

### 3.3 Different Degradation Behaviors in Normal and Inverted Device Structure

In the previous section, the effect of junction formation in normal‐structured device (n‐i‐p) was discussed. Another interesting observation regarding stability of perovskite solar cells was that different patterns in the aging curve (time evolution of PCE) have been quite commonly observed depending on the normal (n‐i‐p) or inverted structure (p‐i‐n). In the case of normal‐structured devices, a sudden, significant drop of the PCE was observed at the beginning of a MPPT test, which is generally referred to as “burn‐in decay”.^[^ ^20d^ ^]^ After this burn‐in degradation, the degradation speed was slower and gradually saturated. This burn‐in degradation seemed to be somewhat reversible in dark (We will discuss more in Section 4.1). Therefore, the degradation behavior observed in normal device structure would have been attributed to ion and defect migration.

The inverted perovskite solar cell with the structure of ITO/PTAA/Mixed perovskite/PS/C_60_/Cu rather experienced the initial efficiency gain at the beginning and there was no clear recovery after resting in the dark.^[^ ^20^, ^81^ ^]^ Inverted perovskite devices seemed to be tolerant of the burn‐in decay, which might probably indicate that there exist no strong electronic band bending (localized e‐field) and ionic motion during MPPT operation could be relatively suppressed than the case for the normal‐structured device having TiO_2_ ETL. Also, hydrophobic materials such as C_60_ and PCBM were deposited on top of the perovskite layer, which may prevent moisture penetration better than the normal device.

## 4 Stabilization Techniques for Working Perovskite Devices

The laboratory‐scale stability test can provide essential information on the lifetime of a solar cell in a controlled situation like stable 1 sun light soaking as well as a fixed humidity and temperature. However, the actual operation in an outdoor circumstance is more dynamic because the amount of sunlight always changes with the time of day, or by the weather condition. First of all, the best practice to stabilize perovskite solar cells and modules will be the robust encapsulation to prevent exposure to oxygen and moisture, and other environmental stresses including rain, snow, wind, and dust. Furthermore, the encapsulation step should consider electrical and mechanical protection for harsh outdoor operation, implying that such packaging is one of the most critical implementations toward commercialization of perovskite solar cells. In this sense, there have been several reviews dedicated to encapsulation and packaging strategies for perovskite devices.^[^ ^82^ ^]^ We can take advantage of a traditional packaging technique using epoxy resin and glass cover which is widely used for commercialized Si and thin film PVs. Also, thin film encapsulation (TFE) techniques employing Al_2_O_3_ or multilayer films can be applied to perovskite devices especially for flexible and low‐temperature processed applications. It is noteworthy that a water vapor transmission rate (WVTR) of 10^−5^ g/m^2^/day is required to effectively prevent moisture‐induced degradation. The TFE techniques can satisfy with the WVTR requirement using various types of protection materials such as Teflon, UV‐curable fluoropolymer, Al_2_O_3_, organosilicate, etc.^[^ ^10b^ ^]^

Second, the real solar panels can have time to recover performance degraded after daytime operation (during nighttime). The PV industry based on Si cells is already taking advantage of the downtime to recover degraded cells caused by potential‐induced degradation (PID).^[^ ^83^ ^]^ As the PID is attributed to a high potential form between a module and the ground, one can recover the PID effect by applying the opposite potential to the module from the ground. This approach is widely used to stabilize solar panels in real market.

The downtime can be even more useful for perovskite solar cells because a couple of degradation mechanisms are found to be reversible when kept in dark.^[^ ^20^, ^84^ ^]^ These reversible characteristics of perovskite materials can be positively exploited. In this section, we review stabilization and healing techniques previously suggested in the literature and discuss a recent advance to stabilize working perovskite devices with no downtime for daytime use.

### 4.1 Reversible Recovery in Dark

**Figure 6:**
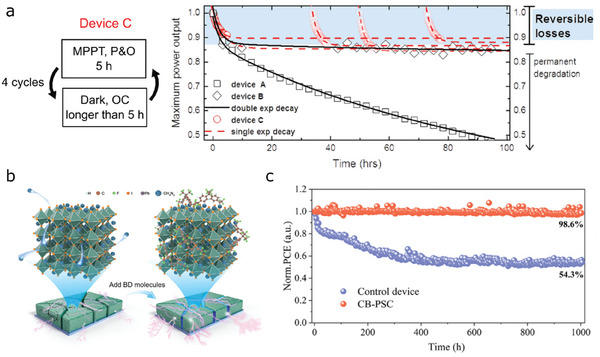
a) a cyclic operation of a perovskite solar cell for enhanced operational stability (left) and time evolution of maximum power output of the cell (red dashed line) operated by the suggested operation cycle. Photovoltaic performance (power conversion efficiency, PCE) decayed during maximum power point tracking (MPPT) was restored after resting the degraded device in the dark condition via ion re‐distribution process, while continuous MPPT led to permanent degradation (black trace and squares). Reproduced with permission from ref. [20a]. Copyright 2015, The Royal Society of Chemistry b) an illustration for a chemical design of perovskite grain boundary to immobilize organic cation using a covalent bonding (CB) molecule. c) time evolution of normalized PCE for a control device (blue symbols) and a CB‐based perovskite device (red symbols) tested under 1 sun illumination in an N_2_ atmosphere. Panels b and c: adapted with permission from ref. [85]. Copyright 2023, Elsevier.

Numerous studies have observed reversible recovery in perovskite solar cells after storing them in the dark condition for overnight, indicating that these devices will have better stability when they are rather tested under outdoor or cycle conditions. As introduced in Section 2.3, these behaviors have been accounted for mainly due to ion‐related motions. The study described in ref. [20a] conducted in‐depth investigations on the reversible behaviors observed in a perovskite solar cell. As shown in **Figure** **6**a, the authors operated the device (named device C) by employing the perturbation & observe (P&O) based MPPT method for 5 h under 1 sun illumination, rested the device in the dark condition for several hours, and repeated 4 cycles of the MPPT and resting steps. The power output as a function of time recorded during these 4 cycles can be seen from the red circles of Figure 6a. Note that the black traces and symbols indicate test results without the resting step. The resting step surprisingly led to almost 100% recovery to its initial power output even if the degradation rate during the MPPT operation was quite rapid (about 10% decrease from the initial power output for 5 hours). On the other hand, the device continuously operated for 100 h showed steady performance decay with no recovery. Reversible losses could be only recovered before permanent degradation occurred due to continuous operation. This indicates a cyclic operation benefitting from nighttime can be utilized to realize the long‐term stable perovskite solar cells.

### 4.2 Immobilization of Organic Cations on Grain Boundaries

It is well established that the degradation occurs at defective grain boundaries as shown in Figure 3d. Liu *et al.* ^[^ ^85^ ^]^ recently reports a covalent bonding strategy to heal the defects that exists at grain boundaries. The authors introduced bis‐diazirine molecules decomposing into carbenes with two unshared valence electrons that enable the covalent bond with organic cations at the grain boundaries (see Figure 6b).^[^ ^86^ ^]^ This covalent bonding process was claimed to immobilize ions at the grain boundaries that can mitigates ion‐ induced instability, therefore, result in much enhanced stability. The authors reported an impressive stability without encapsulation that the power conversion efficiencies maintained 98.6% of the initial efficiency after 1‐sun 1000‐h MPPT operation under an N_2_ atmosphere (shown in Figure 6c).

### 4.3 Pulsatile Therapy for Perovskite Solar Cells

Performance decreases in perovskite solar cells accompany both reversible and irreversible contributions as discussed above. For the long‐term stability, it is of significant importance to take advantage of reversible characteristics, but also required to pay attention to the irreversible part of the degradation. As introduced above, the irreversible degradation took place for a few ten hours of continuous MPPT, meaning that prevention of such irreversible degradation would require special cares within the timeframe of several hours.

**Figure 7:**
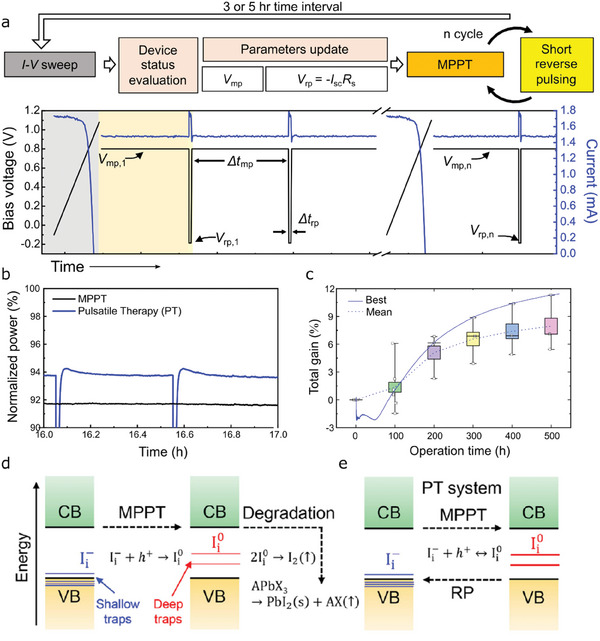
a) Pulsatile therapy (PT) developed for stabilizing working perovskite solar cells without downtime. Photovoltaic parameters obtained from *I*–*V* sweep were used to evaluate the device status and find a maximum power point. A device can be operated by automatic PT algorithm based on the measured parameters, in which short reverse pulses will be applied for a very short time (30 s) during MPPT. An example of current (blue) and voltage (black) profiles during PT operation. b) Normalized power profiles of two devices operated by MPPT (black) and PT (blue), respectively. These two devices were tested at the same time under 1 sunlight soaking. In the case of PT, power was recovered shortly after reverse pulses and its decrease was relatively slower than that of the MPPT‐tested device. c) Total energy gain of the PT‐tested devices as a function of operation time, exhibiting total harvested energy improved by up to 11.3% by PT operation when compared to a standard MPPT operation. d) a schematic illustration of the degradation pathway of perovskite solar cells operated by MPPT. e) a schematic illustration of the healing mechanism of the PT system. Reproduced with permission from ref. [78a]. Copyright 2022, Elsevier.

A recent study presented in ref. [78a] demonstrated a practical method that can improve the device stability by effectively suppressing charge and ion accumulation. In the technique named pulsatile therapy (PT), short reverse pulses were applied to a device working in the middle of MPPT for 30 seconds in order to neutralize a parasitic capacitor induced by charge accumulation and redistribute ion migration. The algorithm of the PT is shown in **Figure** **7**a, together with an example for current and voltage profiles during the PT operation. The authors built an automatic system to execute such an algorithm, in which the device status could be updated for every 3 or 5 h by measuring *I–V* curves. Based on the obtained photovoltaic characteristics, the system uploaded the new PT condition including a MPP and reverse pulse (RP). After the evaluation, the system ran multiple cycles of MPPT (30 min) and short RP (30 sec) until the next *I*–*V* sweep. The conditions were optimized to achieve the best power harvesting with consideration of power consumption during the RP step. Interestingly, the PT method was able to stabilize the tested device by healing the reversible losses shortly after an RP step and slowing down the overall degradation rate as can be seen in Figure 7b. Statistic results for 5 sets tested for continuous 500 h are shown in Figure 7c, presenting that the total energy gain with the help of the PT operation was more than 6% on average. The best case reached up to 11.3% after 500 h, meaning that the PT method led to 11.3% more energy harvesting than the MPPT thanks to the improved operational stability.

The working principle of the PT operation could be described based on the irreversible chemical processes mediated by halide defects. During the MPPT operation, charges and ions will move by the diffusion‐drift model, leading to their localization at interfaces. Positive charges (holes) will attract negatively polarized ions such as I_i_^−^. High concentrations of holes and I_i_^−^ result in the charge‐state transitions to produce the neutral I_i_^0^. (Figure 7d) As already introduced in section 2.5, the neutral I_i_^0^ not only acts as the center of nonradiative recombination, but also causes production of stable species including I_3_^‐^, I_2_, and PbI_2_.^[^ ^26^, ^78^ ^]^ On the other hand, the PT system uses periodic reverse pulses to mitigate accumulation of charges and ions, effectively reducing the concentrations of defects and charges at interfaces. The above‐mentioned charge‐state transition rather occurs reversely due to the reduced concentrations of the reactants. As a result, the degraded photocurrent due to nonradiative recombination occurring at the deep‐level defect can be restored, and the chemical degradation from the neutral I_i_^0^ can be also prevented, leading to healing of device performance and slowing down device degradation. (Figure 7e) The PT technique proposed a novel approach to stabilize a working perovskite solar cell without pause of the operation. Although the technique was not fully optimized, commercialization of perovskite modules could benefit from the PT technique in order to boost their lifetimes and energy gain.

### 4.4 Challenges and Solutions for Perovskite Solar Modules

While the stability of perovskite solar cells has been studied and investigated so far, the long‐term stability of perovskite solar modules has yet to receive much attention despite its importance towards commercialization. Especially, it was experimentally verified that the operational stability of the modules was far poorer compared to that of the small‐area cell with the same device architecture, indicating that there are additional risks in terms of module stability that arise from modulization process.^[^ ^87^ ^]^ First, post‐scribing treatments are required to demonstrate series interconnection between sub‐cells. A scribing method using a picosecond pulsed laser is preferred to reduce heat damages occurring due to the scribing process. However, the amount of energy for laser etching should be high enough to induce local heating, melting, and ablation of perovskite, CTLs, and transparent conducting oxide (TCO), which means that the laser ablation can trigger degradation of the perovskite module and lower the module performance by producing lead iodide (PbI_2_) at the edges of the scribing area.^[^ ^87^ ^]^ Also, incomplete removal of CTLs leads to increasing contact resistances at the interconnections. This not only dramatically lowers the fill factor and PCE of a perovskite module, but also induces accumulated charges or ion migration as a result of inefficient charge extraction.

**Figure 8:**
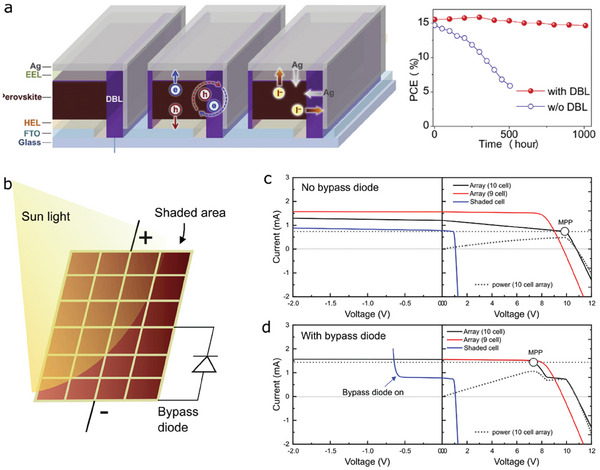
a) A perovskite module architecture employing diffusion barrier layers (DBLs) to prevent charge carrier recombination and ion diffusions. (left) The DBL‐embedded perovskite module showed a dramatic improvement in the operational stability compared to the module without DBL (right) Panel a: reproduced with permission from ref. [89]. Copyright 2019, Elsevier. b) an illustrative example of a perovskite panel working in a shaded area. Implementation of a bypass diode improves energy harvesting and operational stability in case unwanted shading exists due to clouds, dusts on the panels, and other external issues. c) *J–V* simulation for a 10‐cells array when it is assumed that photo‐generated current is reduced by half due to shading. Current output of the entire array is limited to the maximal current generation of the shaded cell (see the black line). The unshaded 9‐cells array lose its performance and experience high forward bias due to the shading on the other cell. d) *j‐V* simulation for a 10‐cells array with bypass diodes, allowing for the stable MPPT operation with a high PCE. The system enables to generate high current output from the unshaded 9 cells by turning on the bypass diode connected to the shaded cell in parallel. Panels c,d: reproduced with permission from ref. [78a]. Copyright 2022, Elsevier.

Additionally, the post‐etching step creates more interfaces and areas exposed to air or metal electrodes.^[^ ^88^ ^]^ Surfaces uncovered by etching are possibly damaged due to heat or chemicals used in the etching steps, which may be more subject to unwanted ion migration and irreversible chemical reactions with air or metal ions. According to a study presented in ref. [89], the increased areas exposed to metal electrodes and air provided additional routes for ion diffusion and charge carrier recombination. (**Figure** **8**a, left) As a result, operational stability of the module was fairly poor. (Figure 8a, right, blue line and symbols) The author of the study performed special treatment to protect the uncovered interfaces by adding diffusion barrier layers comprising low‐dimensional materials, which led to a noticeable improvement in the operational stability of the treated module (Figure 8a, right). The module maintained more than 90% of its initial efficiency after 1000 h under 1 sun light soaking.

Another impact in a module may come from performance non‐uniformity between sub‐cells. In the case of series interconnect, the current output from a module is limited by the worst‐performing sub‐cell, causing unbalanced applied biases to sub‐cells for MPPT operation. The well‐working sub‐cells should be situated at a higher bias, possibly leading to a faster degradation as explained in Section 3.1. Therefore, a high uniformity of performance between sub‐cells allows one to achieve better stability in a module. An unavoidable situation to risk module operation is illustrated in Figure 8b, which is due to unwanted partial shading (for example, outdoor operation in a cloudy weather, or dirt accumulated on a solar panel can cause shading). The partial shading not only leads to huge performance losses but causes irregular electrical biases on sub‐cells like even reverse biasing to a shaded cell.^[^ ^31^, ^90^ ^]^ In particular, reverse current densities become as high as a few 100 mA cm^−2^ in the presence of high reverse biases (>10 V), generating considerable heat energy and local hot‐spot.^[^ ^90c^ ^]^ Such effects further lead to heat‐induced degradation and severe ion migration, thereby lowering module performance and stability.^[^ ^31^, ^90^ ^]^ To tackle the shading‐induced issue, one can take advantage of implementation of bypass diodes connected in parallel to a sub‐cell. As a bypass diode allows for current flowing even if there is no photocurrent output in a connected cell, photocurrent from other sub‐cells flow smoothly through the bypass diode. An exemplary *I‐V* modeling for a module connected with bypass diodes is shown in Figure 8d, in which it can be confirmed that stable MPPT operation is available even if a sub‐cell is 50% shaded. Implementation of bypass diodes is compatible with pulsatile therapy introduced in Section 4.2. Therefore, it can be practically utilized to stabilize perovskite modules operated any operational method.

## 5 Summary and Outlook

It has been more than a decade since perovskite solar cells emerged as potential alternative of conventional solar devices. The field has made huge progress with respect to photovoltaic performance, long‐term stability, fabrication methods, modulization, etc. The PCE of perovskite single‐junction solar cell almost ties that of the best Si solar cell.^[^ ^1^, ^2^ ^]^ More surprisingly, perovskite/Si tandem devices set a new record overcoming the Shockley–Queisser limit for Si solar cells.^[^ ^3^ ^]^ The commercialization of perovskite‐based devices seems like a matter of time. However, the level of PCE demanded for practical implementation is in fact not that high as the most commercial solar panels have PCEs ranging from 15% to 20%.^[^ ^91^ ^]^ Since these values are already realizable with perovskites, the real market will demand comparable lifetimes to compete with conventional solar price per kilowatt‐hour (typically $0.06/kWh).^[^ ^11^ ^]^

When it comes to the long‐term stability, there have also been many milestones including demonstration of solid‐state perovskite solar cells, two‐step spin‐coating techniques, solvent, and compositional engineering, low‐dimensional (2D, quasi‐2D, and 2D/3D^[^ ^92^ ^]^ perovskites. These advances have been mainly aimed to dramatically improve photovoltaic performance from material engineering, but they always accompany significant increases in their long‐term stability. In other words, the advances in performance had been coupled to the improvement in stability because trap (defect) states and nonradiative recombination play critical roles in both performance and stability. In that sense, single‐crystal perovskite solar cells are one of compelling candidates for achieving year‐scale lifetime.^[^ ^87^ ^]^ Moreover, stain engineering needs to be explored further in order to realize high material stability by suppressing ion migration and segregation, and the influence of cracks and wrinkles in the perovskite film should be also taken care.^[^ ^87^ ^]^ Such material innovations must continue to accelerate practical implementation.

Along with material engineering for solving the stability issue, much effort has also been made to scientifically prove degradation mechanisms and their origins depending on different degradation circumstances. All these studies have pointed out that ion migration and trapped charges are mainly responsible for perovskite degradation. The degradation can be accelerated in the presence of environmental hazards such as moisture, oxygen, and heat especially where their synergetic effects actively trigger irreversible chemical reactions. Even though degradation pathways are much complicated and dissimilar depending on the conditions, all the pathways are accelerated when those two main origins (ion migration and trapped charges) are developed in a device. That is, a perovskite solar cell working under light illumination and electrical bias shows more unstable features than a resting device even at high temperature and high humidity.

The operational stability represented by on a suggested stability test protocol (ISOS‐L) has also been one of research goals in tackling the stability issues. One‐year operational stability was realized for the first time in 2017,^[^ ^13a^ ^]^ but unfortunately, reported lifetimes in recent stability‐related studies still stays the similar level likely due to the fact that it is not realistic to do year‐scale stability test for publication. The accelerated ageing test such as ISOS‐L‐3 (1 sun, 85°C, RH∼50%) is required to evaluate device lifetime for a relatively short period of time using a correct acceleration factor.

Degradation studies for working perovskite solar cells have revealed that both charge and ion accumulations at interfaces induce irreversible chemical reactions mediated by deep‐level defects. Electronic band bending at a heterointerface also plays a crucial role in causing accumulation of charges and ions due to the localized electric field, leading to fast deterioration of perovskite morphologies. In addition, the level of an electrical bias applied to a device strongly affects the amount of charge accumulation. The devices operated at a high bias (e.g. MPP and OC) reproducibly showed faster degradation than the devices operated at a lower bias where charges were extracted with no electrical hindrance. This may indicate that an operation at an optimized bias is possibly better than a standard MPPT when it comes to the long‐time operation.

Covalent bonding strategy has been recently developed to immobilize the organic cations on grain boundaries that could prevent ion migration and eventually greatly increase the operation stability of perovskite solar cells. A cyclic operation mimicking an actual operation can stabilize perovskites through ion redistributions and release of trapped charges during nighttime. Similarly, a recent advance to stabilize working perovskite solar cells is pulsatile therapy, in which reverse pulses are applied for a short time to return ions and charges in the middle of a standard MPPT. The technique brings two efficacies for long‐term stability: reversible healing of degraded performance and deceleration of degradation during MPPT. It can be also expected that the combination of a low‐bias energy‐harvesting and the pulsatile therapy demonstrate unprecedented operational stability over a few years when they are applied to a stable perovskite solar cell comprising an ideal p‐i‐n junction.

## Conflict of Interest

The authors declare no conflict of interest.
